# Cardiolipin Structure and Oxidation Are Affected by Ca^2+^ at the Interface of Lipid Bilayers

**DOI:** 10.3389/fchem.2019.00930

**Published:** 2020-01-21

**Authors:** Érica G. A. Miranda, Juliana C. Araujo-Chaves, Cintia Kawai, Adrianne M. M. Brito, Igor W. R. Dias, Jeverson T. Arantes, Iseli L. Nantes-Cardoso

**Affiliations:** ^1^Laboratory of Nanostructures for Biology and Advanced Materials, NanoBioMAv, Center of Natural Sciences and Humanities, Federal University of ABC, Santo André, Brazil; ^2^Center of Engineering, Modeling, and Applied Social Sciences, Federal University of ABC, Santo André, Brazil

**Keywords:** singlet oxygen, calcium, cardiolipin, free radicals, GUVs, lipid packing, oxidative stress

## Abstract

Ca^2+^-overload contributes to the oxidation of mitochondrial membrane lipids and associated events such as the permeability transition pore (MPTP) opening. Numerous experimental studies about the Ca^2+^/cardiolipin (CL) interaction are reported in the literature, but there are few studies in conjunction with theoretical approaches based on *ab initio* calculations. In the present study, the lipid fraction of the inner mitochondrial membrane was modeled as POPC/CL large unilamellar vesicles (LUVs). POPC/CL and, comparatively, POPC, and CL LUVs were challenged by singlet molecular oxygen using the anionic porphyrin TPPS4 as a photosensitizer and by free radicals produced by Fe^2+^-citrate. Calcium ion favored both types of lipid oxidation in a lipid composition-dependent manner. In membranes containing predominantly or exclusively POPC, Ca^2+^ increased the oxidation at later reaction times while the oxidation of CL membranes was exacerbated at the early times of reaction. Considering that Ca^2+^ interaction affects the lipid structure and packing, density functional theory (DFT) calculations were applied to the Ca^2+^ association with totally and partially protonated and deprotonated CL, in the presence of water. The interaction of totally and partially protonated CL head groups with Ca^2+^ decreased the intramolecular P-P distance and increased the hydrophobic volume of the acyl chains. Consistently with the theoretically predicted effect of Ca^2+^ on CL, in the absence of pro-oxidants, giant unilamellar vesicles (GUVs) challenged by Ca^2+^ formed buds and many internal vesicles. Therefore, Ca^2+^ induces changes in CL packing and increases the susceptibility of CL to the oxidation promoted by free radicals and excited species.

## Introduction

Mitochondria are vital organelles for several metabolic processes involved in cellular function and dysfunction. Mitochondria participate in cell cycle control, growth, differentiation, and play an essential role in Ca^2+^ homeostasis (Area-Gomez et al., 2019; Madreiter-Sokolowski et al., 2019; Woods et al., 2019). For mitochondria, the consequence of placing the oxidative metabolism is the primary generation of superoxide ion that leads to the formation of secondary pro-oxidant species such as hydrogen peroxide, peroxynitrite, hydroxyl radicals, carbon-centered radicals, triplet carbonyls, and singlet oxygen (Misztal et al., 2013; Miyamoto et al., 2014; Prasad et al., 2018; Radi, 2018; Ucar et al., 2019). Mitochondrial oxidative and nitrosative stress is implicated in degenerative and cardiovascular diseases (Liguori et al., 2018). A well-known condition leading to the increase of superoxide production in mitochondria is the ischemia and reperfusion of tissues such as the myocardium and are events associated with the opening of the mitochondrial permeability transition pore (MPTP) (Kalogeris et al., 2014; Kim-Campbell et al., 2019; Menezes-Filho et al., 2019; Neginskaya et al., 2019; Sarkar et al., 2019). MPTP is an unspecific pore for low molecular mass molecules (≤1.5 kDa) inhibited by cyclosporine A (Giorgio et al., 2018; Vercesi et al., 2018). MPTP opens in Ca^2+^-overloaded mitochondria leading to mitochondrial swelling and depolarization, inhibition of oxidative phosphorylation and stimulation of ATP hydrolysis (Zhao et al., 2004; Clarke et al., 2008; Giorgio et al., 2018; Vercesi et al., 2018; Xie et al., 2019). Studies about MPTP gained high relevance due to the participation of this pore in both apoptotic and necrotic cell death (Kim-Campbell et al., 2019). The role of Ca^2+^ in the MPTP opening has been associated with the interaction with proteins and phospholipids of the inner mitochondrial membranes (IMM) (Nantes et al., 2011; Vercesi et al., 2018). In 1988, Crompton and Costi (1988) reported that cyclosporine A could inhibit the opening of a Ca^2+^-dependent pore in heart mitochondria challenged by Ca^2+^ plus tertbutyl hydroperoxide. The studies about cyclosporine A also linked MPTP opening to oxidative stress and ischemia-reperfusion injury (Griffiths and Halestrap, 1993; Qian et al., 1997). Ischemia promotes an increase of the intracellular Ca^2+^ to levels enough to cause mitochondrial Ca^2+^ overload upon reoxygenation (Halestrap, 2006; Vercesi et al., 2018). The effect of Ca^2+^ has been related to the production of membrane defects (Fagian et al., 1990; Turin et al., 1997; Kowaltowski et al., 1998) and its binding is favored by the exclusive presence of CL (~20% of total lipids) in the IMM. CL is associated with Ca^2+^ binding and modulates the structure and function of the respiratory complexes and cytochrome c (Powell et al., 1987; Hoch, 1992). The binding and effects of Ca^2+^ on the biological membranes have been extensively investigated by experimental studies (Pessoto et al., 2015). In a minor extension, CL has been the object of theoretical studies. Dahlberg et al. (2010) presented *ab initio* Density Functional Theory (DFT) calculations of the cardiolipin (CL) headgroup and its 2′-deoxy derivative (dCL). The authors estimated that protons could exchange between phosphate groups with an energy barrier of ~4–5 kcal/mol. Lemmin et al. stated that in-depth models and characterization of cardiolipins are to date rare and proposed an ab initio parametrization of cardiolipin species for molecular simulation and concluded that the cardiolipin protonation influences the lipid packing (Lemmin et al., 2013). The authors also investigated the interactions with Na^+^ and Mg^2+^ but not Ca^2+^. To the best of our knowledge, the literature lacks a conjugated theoretical and experimental study about the effect of Ca^2+^ and water in the whole CL structure. In the present study, it is theoretically demonstrated that Ca^2+^ affects CL structure influenced by the protonation state of the lipid headgroup. Also, experimental studies showed that the effects of Ca^2+^ on the CL-containing lipid bilayer organization increased its susceptibility to the oxidation by singlet oxygen and free radicals.

## Results

**Figure 1:**
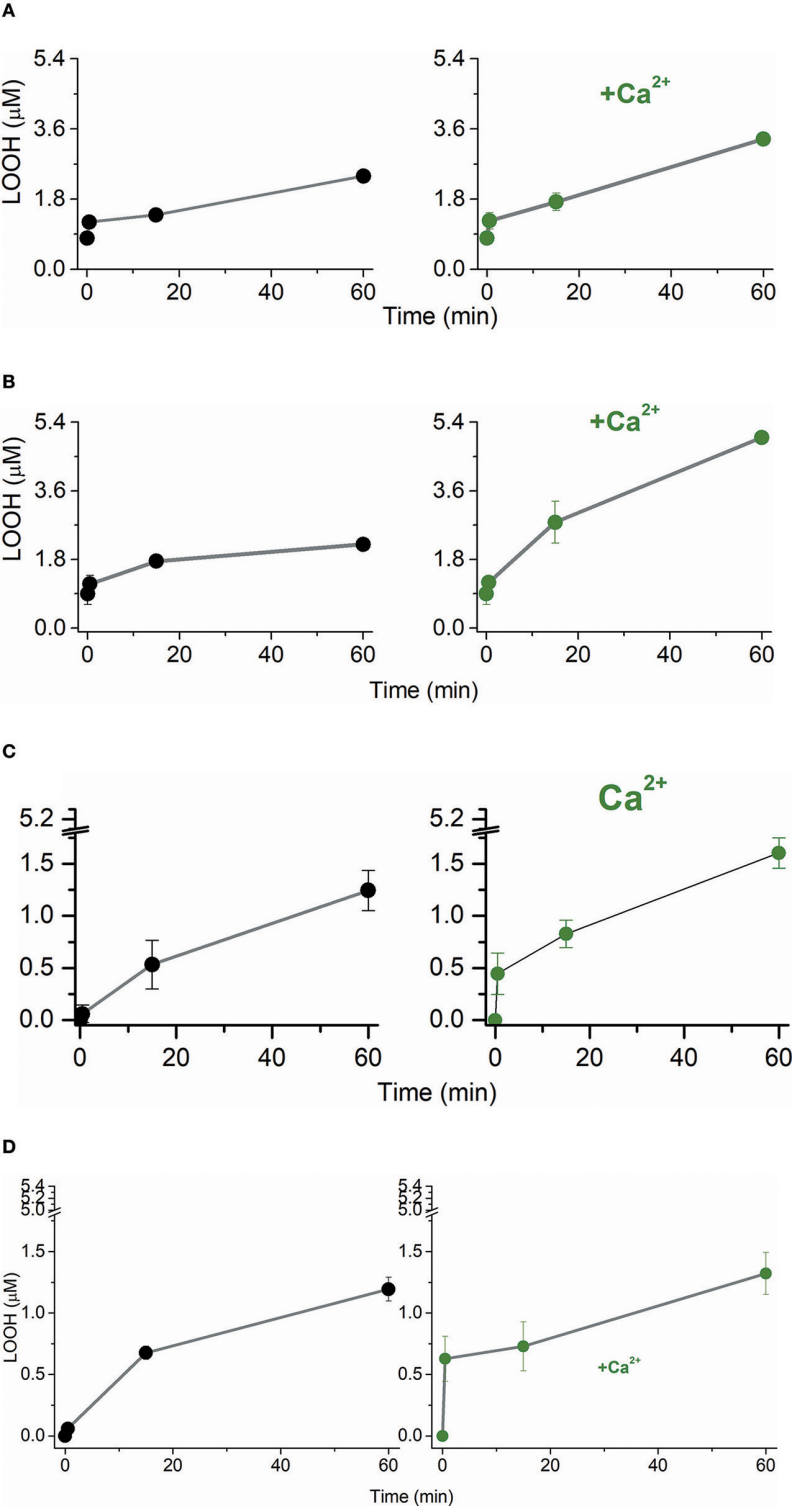
Effect of Ca^2+^ on the LOOH production by singlet oxygen and free radicals in phospholipid bilayers. **(A)** Temporal LOOH production by singlet oxygen in POPC/TOCL LUVs in the absence (left, black line, and symbol) and presence (right, olive line, and symbol) of 3 mM Ca^2+^. **(B)** Temporal LOOH production by singlet oxygen in POPC LUVs in the absence (left, black line, and symbol) and presence (right, olive line, and symbol) of 3 mM Ca^2+^. **(C)** Temporal LOOH production by singlet oxygen in TOCL LUVs in the absence (left, black line, and symbol) and presence (right, olive line, and symbol) of 3 mM Ca^2+^. **(D)** Temporal LOOH production by Fe^2+^/citrate in BBCL LUVs in the absence (left, black line, and symbol) and presence (right, olive line, and symbol) of 3 mM Ca^2+^. In **(A–C)**, singlet oxygen was generated by the irradiation of 10 μM of TPPS4 using LED array that emits 200 μW/cm^2^ in 532 nm. In **(D)**, polyunsaturated BBCL was oxidized by 50 μM Fe^2+^/citrate as described in Materials and Methods. The experiments of oxidation were performed in deionized aqueous solution of 0.2 M of glucose, pH = 6.5, at 25°C.

**Scheme 1:**
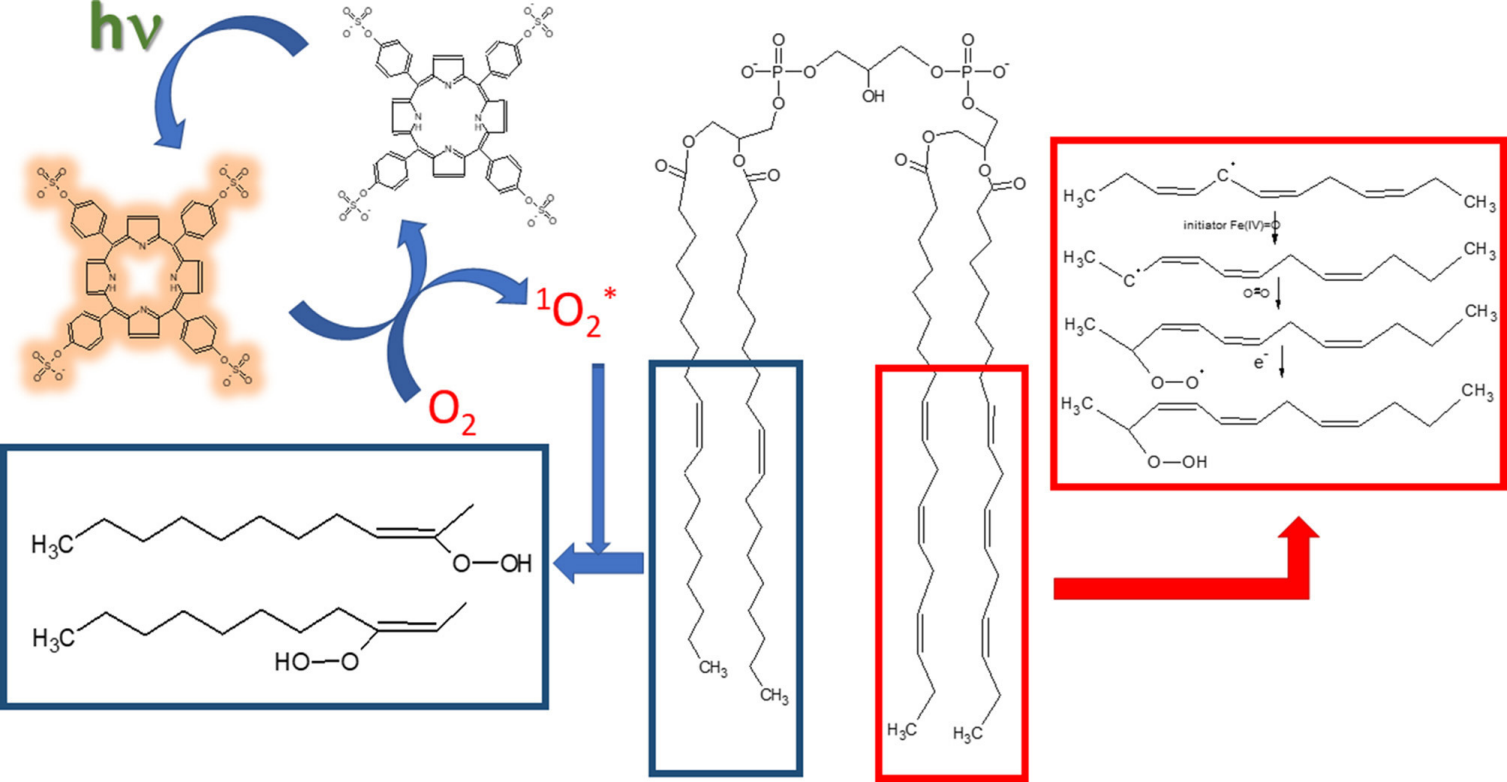
Mechanisms of lipid oxidation by singlet oxygen and Fe^2+^. The structures inside blue-bordered box refer to oxidation promoted by singlet oxygen and the structures inside red-bordered box shows the oxidation by free radicals promoted by Fe^2+^. Porphyrin structure with brownish yellow glow represents the molecule in the triplet excited state.

The Ca^2+^-promoted changes in CL structure and packing might affect the susceptibility of lipid membranes to the attack of free radicals to an allylic carbon and the addition of singlet oxygen to the double bonds of unsaturated acyl chains leading to the formation of lipid peroxides (LOOH). Considering this premise, we modeled the lipid fraction of the inner mitochondrial membrane arranged as POPC (1-palmitoyl-2-oleoyl-sn-glycero-3-phosphocholine)/TOCL (tetraoleoyl cardiolipin) large and giant unilamellar vesicles (LUVs and GUVs, respectively). The PC and CL fractions of the lipid bilayers were also analyzed individually by using POPC and TOCL LUVs. TOCL LUVs were replaced by BBCL (bovine brain cardiolipin) LUVs when Fe^2+^/citrate was the pro-oxidant agent. LUVs were challenged by the oxidative attack of excited species and free radicals simulating the events occurring in photodynamic therapy (PDT) and chemical oxidative stress promoted by the Fenton reaction. A PDT model was constructed using the anionic porphyrin TPPS4 (meso-tetrakis(4-sulfonatophenyl) porphyrin) as the photosensitizer for the generation of singlet molecular oxygen via energy transfer to ground state molecular oxygen. TPPS4 irradiated by a LED array was the singlet oxygen source for POPC/TOCL (80/20 mol%), POPC (100%) and TOCL (100%). TPPS4 was chosen because this molecule does not have an affinity to negatively charged interfaces (Kawai et al., 2014a). Therefore, we excluded the interference of porphyrin binding on the susceptibility of phospholipids to oxidative damages promoted by the excited species. In this model, the singlet oxygen attack to lipids could be determined by LOOH formation (Bacellar et al., 2018). The model of chemical oxidative stress was constructed using Fe^2+^-citrate targeting the lipid peroxides of the membranes (Pessoto et al., 2009). POPC/CL large unilamellar vesicles (LUVs) were challenged by singlet oxygen produced by the irradiation of TPPS4 added to the medium (Figure 1A). The presence of 3 mM Ca^2+^ increased LOOH production at late times of irradiation. Considering that LUVs have a mixture of POPC and TOCL, it was investigated the specific effect of 3 mM Ca^2+^ on each type of lipid used (Ohyashiki et al., 2002; Rodrigues et al., 2007). Therefore, POPC and TOCL LUVs were separately challenged by singlet oxygen (Figures 1B,C). Like observed for POPC/TOCL vesicles, Ca^2+^ increased the production of LOOH in POPC LUVs, at late times of irradiation. For TOCL LUVs, Ca^2+^ increased both the rate and the yield of LOOH produced by PDT, in comparison with the same membrane in the absence of the ion. In this condition, most of the oxidized lipid was formed at the early times of irradiation (Figure 1C). The presence of Ca^2+^ also increased the rate of LOOH production in BBCL LUVs challenged by Fe^2+^-citrate (Figure 1D). In this case, BBCL was used because the oxidative mechanism of the Fenton reaction requires the presence of an allylic carbon in a polyunsaturated acyl chain (Scheme 1). It was observed a higher total LOOH production for POPC vesicles in the presence of Ca^2+^ than for CL-containing vesicles (Figures 1A,C,D). This result could be due to a better membrane vulnerability to the oxidative attacks induced by a less packed membrane (Khalifat et al., 2011). Differences in the kinetics and yield of LOOH production in POPC and POPC/TOCL membranes are consistent with previous studies (Ahmed-Adrar et al., 2009) that demonstrated a strong protective effect of CL against PC peroxidation that was observed at the range of 5–15% of CL. The assignment of the apparent protective effect of CL on POPC exclusively to a higher sensitivity this lipid to oxidation can not explain the lower total yield of LOOH in membranes of 100% TOCL. In the case of LOOH production by singlet oxygen, it is important to consider the physical quenching of the excited species by lipid acyl chains (Krasnovsky et al., 1983; Vever-Bizet et al., 1989). Literature data reports that the oxidation of CL promotes conformational change both in the backbone/head group and in acyl chain of the lipid (Vähäheikkilä et al., 2018). The polar groups formed in oxidized lipids such as LOOH move to regions closer to the membrane–water interface and contributes to a higher area per lipid chain as well as a decrease of membrane thickness. These structural changes could influence the physical quenching of singlet oxygen by acyl chains. Therefore, in CL-containing membranes, a rapid accumulation of LOOH occurs at the early times of irradiation. However, specially if the physical quencher is favored in oxidized CL membranes, although the faster rate of oxidation, the total yield of LOOH should be lower in these membranes. This is a possible mechanism that requires future investigations. Thus, the affinity of Ca^2+^ to CL affecting the lipid structure and making it more susceptible to oxidation is consistent with a higher and faster LOOH production observed in CL-containing membranes compared with the same membrane where Ca^2+^ was absent. The comparative analysis of the differences in the total LOOH yield observed in different membranes should involve other mechanisms to be elucidated in the future.

Scheme 1 depicts the mechanisms of LOOH production by singlet oxygen and by Fe^2+^/citrate. In the photochemically-induced lipid oxidation, a sensitizer such as porphyrin is promoted to the singlet excited state by the light absorption and converted to the triplet excited state via intersystem crossing (ISC). In aerated media, the triplet sensitizer is deactivated by energy transfer to molecular oxygen, which, in turn, is converted to singlet molecular oxygen (1Δg). Singlet molecular oxygen adds to a double bond of the acyl chain and produces the peroxide derivatives. The lipid oxidation promoted by Fe^2+^/citrate is initiated by the formation of oxyferryl species (Fe(IV) = O) in the presence of Fe^3+^ (Ohyashiki et al., 2002). Oxyferryl abstracts one electron of an allylic carbon leading the formation of conjugated dienes. The carbon-centered radical reacts with molecular oxygen and produces the lipid peroxyl radical prone to attack another allylic carbon that leads to the propagation of the lipid oxidation. Several other oxidized products can be formed in the process of lipid oxidation promoted by singlet oxygen and Fe^2+^/citrate. However, the focus was the effect of Ca^2+^ on the lipid oxidation, and LOOH production was used as a molecular marker of lipid oxidation.

Despite the differences in the oxidative mechanisms involved in photochemical and chemical oxidations, Ca^2+^ favored the pro-oxidant effects of electronically excited and free radical species. Ca^2+^ had peculiar effects in the oxidation kinetics according to the lipid composition, independently of the oxidative mechanism. These results are consistent with the effect of Ca^2+^ on the lipid bilayer packing that modulates the accessibility of the acyl chain double bonds to the attack of pro-oxidant species. It was performed DFT calculations for the lipid interaction with Ca^2+^ and water molecules to investigate the changes promoted by Ca^2+^ on the CL structure with repercussion on the lipid bilayer packing. DFT calculations were preceded by FTIR analysis of the CL at pH 2 and 6.5 to access the protonation state of the lipid.

**Figure 2:**
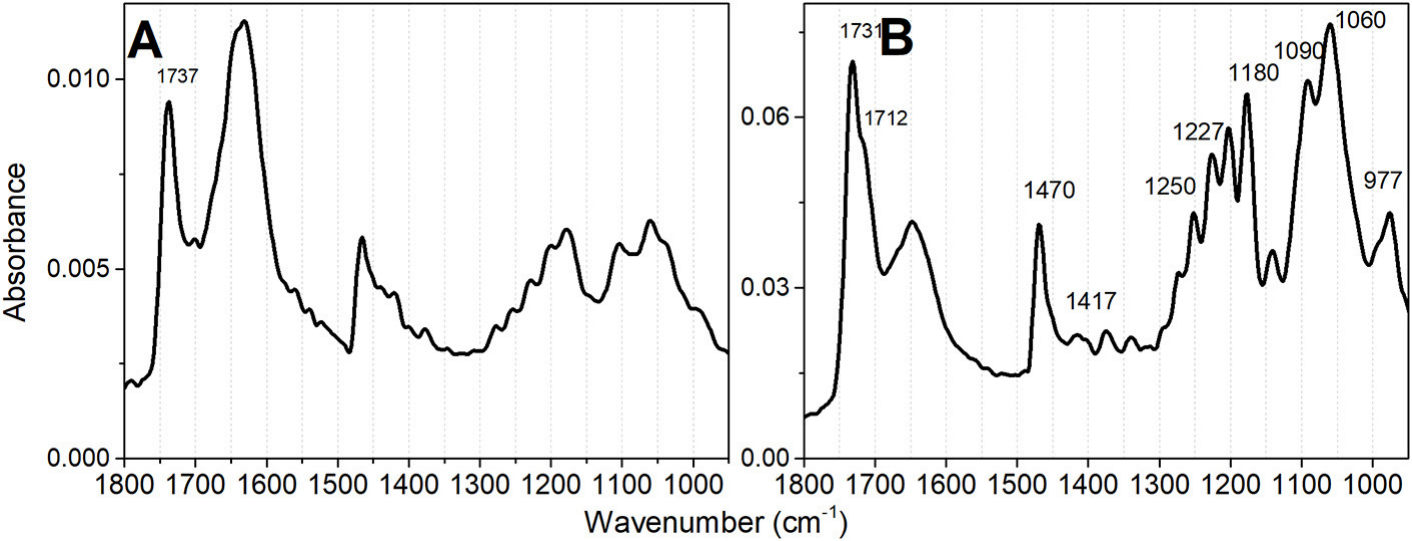
Infrared absorbance spectra of TOCL vesicles at different pH values. **(A)** Spectrum obtained at pH 2 and **(B)** Spectrum obtained at pH 6.5. The lipid concentration was 5 mM in deionized water. The desired pH was obtained by lipid titration.

The features of infrared spectra of lipids are influenced by the degree of hydration, the structural organization, and specific hydrogen bonding influenced by the protonation states of the phosphate head groups (Lewis et al., 1994; Lewis and McElhaney, 2000; Kooijman et al., 2017). CL has two phosphate groups and a complex ionization behavior, which has been thoroughly studied in different conditions and by a diversity of techniques. Most studies are consistent with CL as being fully deprotonated at physiological pH and rest on a single p*K*_a_ value for both phosphate groups of the lipid (Blume et al., 1988; Lewis et al., 1994; Lewis and McElhaney, 2000; Kooijman et al., 2017). In the minority, some studies have demonstrated two widely separated p*K*_a_ values (Hübner et al., 1991; Kates et al., 1993; Gorbenko et al., 2006; Hielscher et al., 2009) and include the theoretical study of Dahlberg et al. (2010) and Lemmin et al. (2013). It is not the main objective of the present study to approach the question of cardiolipin p*K*_a_ values. Therefore, the lipid samples were analyzed by FTIR at pH values of 2.0 and 6.5 to assure that CL was not totally protonated at the experimental conditions of lipid oxidation (pH 6.5). The pH 6.5 corresponds to the expected value at the interface of the outer side of the inner mitochondrial membrane in the energized state of the organelle, i.e., with the transmembrane potential (ΔΨ) generated by the proton gradient in a coupled mitochondrion (Kawai et al., 2013). Different features observed in the FTIR spectra at pH 2 and 6.5 warrant that CL was not totally protonated at pH 6.5. Even in liposomes with mixed lipids, in the absence of proteins, cardiolipin forms domains (Kawai et al., 2014b) and is reasonable to consider that singly and doubly unprotonated CL molecules may be present in these lipid bilayers. In Figure 2, the organization of CL as vesicles, at pH 6.5 is indicated by the sharp band peaking at 1,732 cm^−1^ with a shoulder at 1,718 cm^−1^ that is assigned to the C = O ester group. The CL FTIR spectrum obtained at pH 2 (Figure 2A) presents low-intensity signals of γ CH2 wagging vibrations, i.e., the Snyder modes of CH_2_ groups, between 1,400 and 1,150 cm^−1^. The Snyder modes of CH_2_ groups are intensified at pH 6.5 (Figure 2B), with a sharp band in 1,732. The contributions of symmetric and asymmetric vibration of the P = O and the CO–PO functional groups are in the spectral region of 1,250–840 cm^−1^ (Mantsch et al., 1981; Mendelsohn et al., 1984; Mantsch and McElhaney, 1991; Guan et al., 1994).

**Figure 3:**
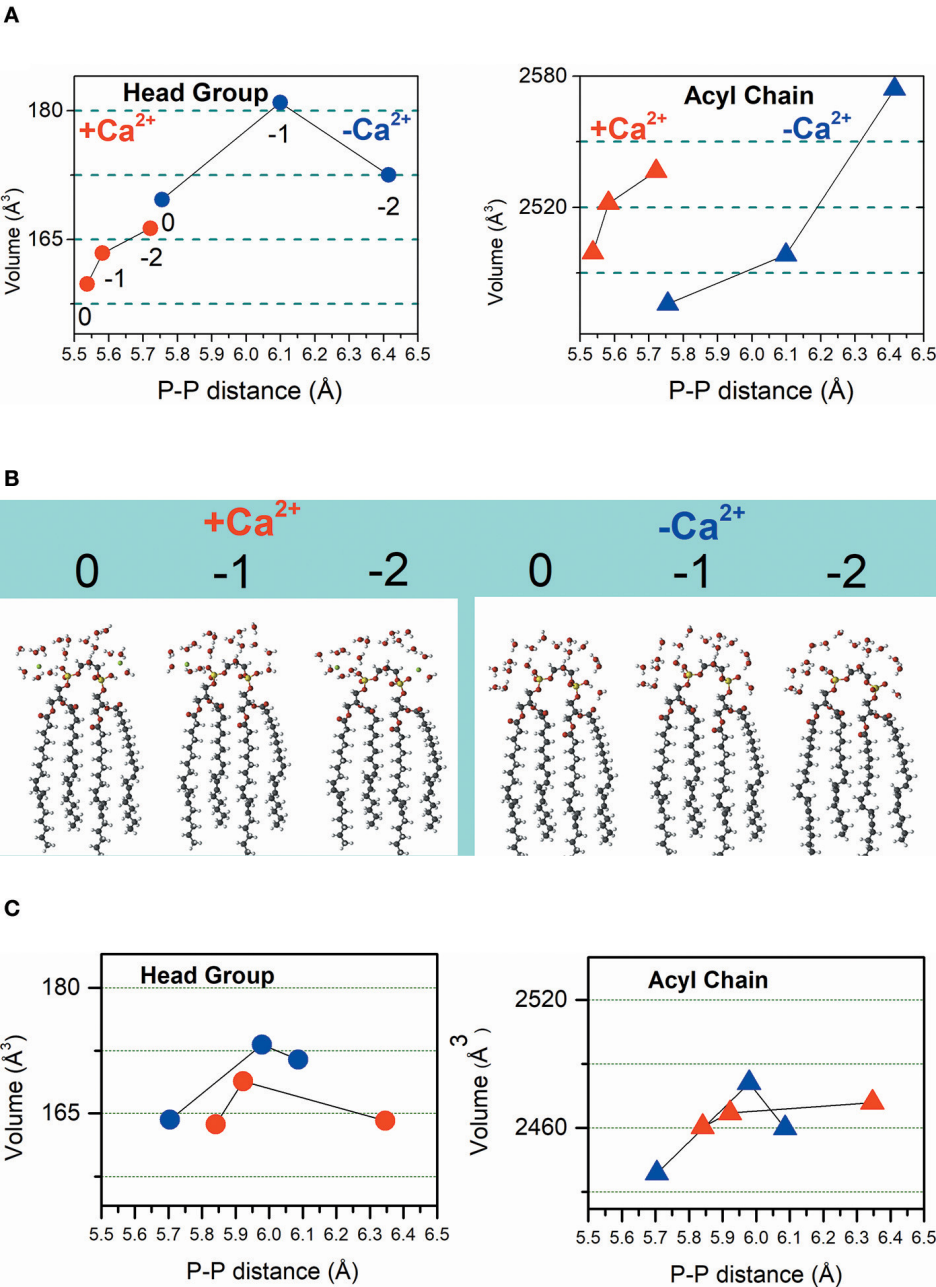
Calculated volumes of the CL headgroup and acyl chains associated with phosphate-phosphate distance influenced by the protonation degree and hydration in the absence (–Ca^2+^) and the presence (+Ca^2+^) of the ion. Dimensions of head groups are represented by solid circles and dimensions of acyl chains are represented by solid triangles. **(A)** Hydrated CL. Symbols in red represent results obtained in the presence of Ca^2+^, and the symbols in blue represent the results achieved in the absence of Ca^2+^. **(B)** The inset shows the calculated CL structures with head group charges of 0, −1, and −2 in the presence of Ca^2+^ (left) and the absence of Ca^2+^ (right). **(C)** Non-hydrated CL. In this panel, symbols in red represent results obtained in the presence of Ca^2+^ and the symbols in blue represent the results achieved in the absence of Ca^2+^. From left to right the CL head groups have charges charges of 0, −1, and −2 that is noted only in the left panel in **(A)**, for clarity.

For the theoretical calculations, it was necessary to consider all the protonation states of CL and the species with head charges of 0, −1, and −2 were calculated. The DFT calculations were performed for CL structure by combining hydration in the absence and the presence of Ca^2+^ with three protonation degrees of CL, i.e., protonated, partially and totally deprotonated states that were noted as 0, −1, and −2, respectively (Figure 3A). To the notation above, –Ca^2+^ and +Ca^2+^ were included in conditions in which the ion is absent and present, respectively.

**Figure 4:**
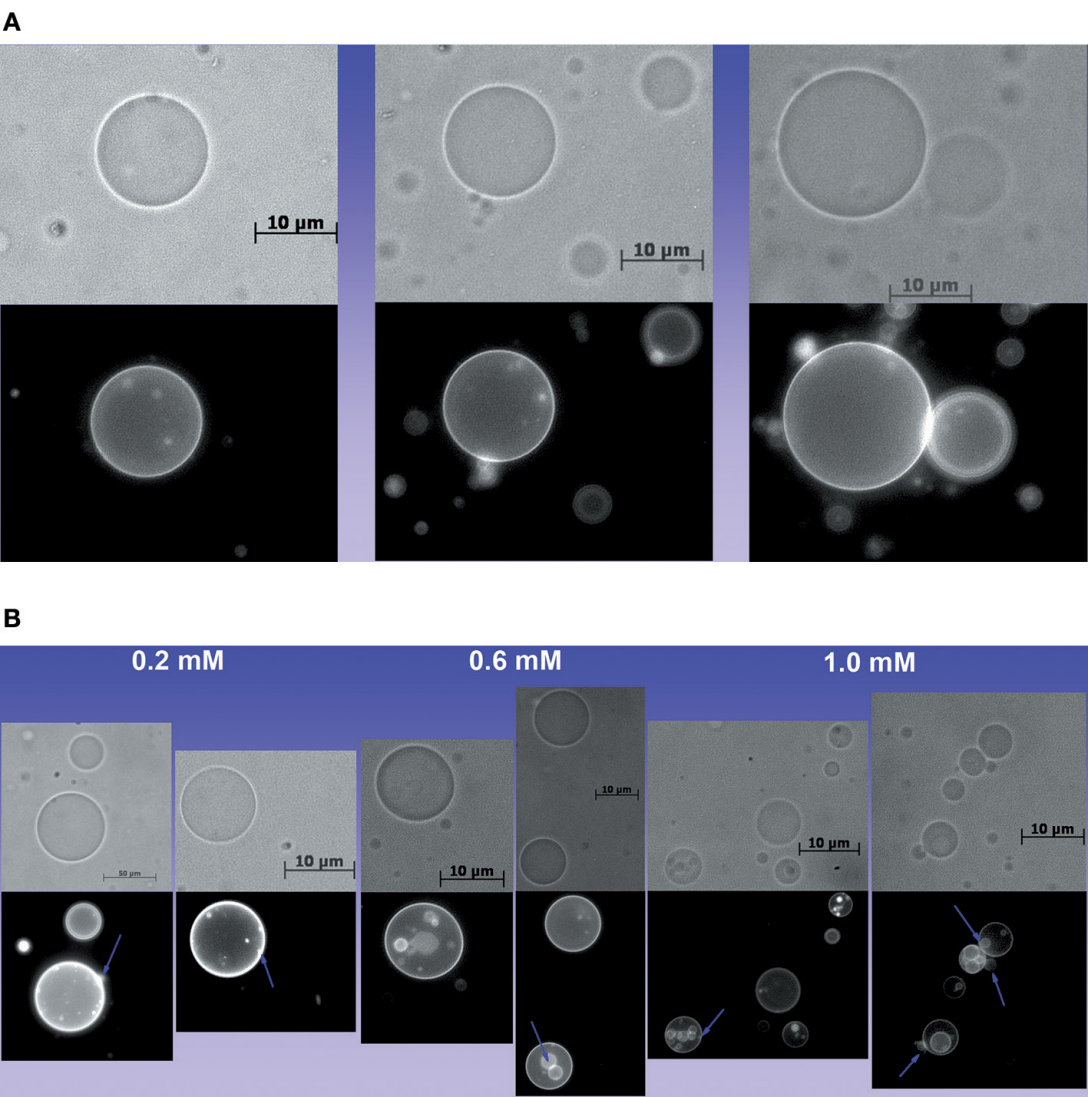
Effect of Ca^2+^ on the structure of GUVs. **(A)** Images of POPC/TOCL GUVs in the absence of Ca^2+^, **(B)** Images of GUVs treated with 0.2, 0.6, and 1.0 mM of Ca^2+^ in which lamellas and buds are evident. Some buds and lamellas are indicated by violet arrows.

The results of DFT calculations presented in Figure 3A were performed for hydrated CL. Consistently, Figure 3A shows that in the absence and presence of Ca^2+^, deprotonation increases the P-P distance. The interaction of CL head groups with Ca^2+^, in all the three protonation states, decreases the intramolecular P-P distance relative to the head group in the corresponding protonation state, significantly. The interaction with Ca^2+^ increases the hydrophobic volume of the acyl chains of protonated and partially deprotonated CL relative to the CL in the corresponding protonation state. Deprotonated CL had a decrease of acyl chain volume in the presence of Ca^2+^. Ca^2+^ propitiated a combination of decreased head group volume with increased acyl chain volume. Figure 3B shows the calculated hydrated structures. Calculations were also performed for non-hydrated CL to get information about the relevance of headgroup hydration on the effects of Ca^2+^ (Figure 3C). Figure 3C shows that the effects of Ca^2+^ on CL structure are significantly different in the absence of water. The decrease of P-P distance was observed only for partially protonated CL; however, in this condition, the increase of headgroup volume was not accompanied by increasing of acyl chain volume. Theoretical calculations predicted that the changes induced by Ca^2+^ in CL structure should affect membrane stability. The theoretically predicted effect of Ca^2+^ on hydrated CL structure was reinforced by an additional experimental study using giant unilamellar vesicles (GUVs) of POPC/TOCL at pH around 6.5. The POPC/TOCL GUVs labeled with fluorescent CL (TopFluorCL) were challenged by Ca^2+^ and analyzed by fluorescence microscopy (Figures 4A,B).

Figures 4A,B show that Ca^2+^ induced the formation of buds and many internal vesicles in the GUVs, some of them are indicated by violet arrows in Figure 4B. This result is consistent with the effect of Ca^2+^ in the lipid packing (Grijalba et al., 1999; Van Meer et al., 2008; Graber et al., 2017).

## Discussion

Mitochondria are organelles that place a wide variety of cellular metabolic processes, including the production of ATP and the buffering of cytosolic calcium. In cell conditions in which there is a disruption of the Ca^2+^ homeostasis, mitochondria uptake and retain Ca^2+^ trying to restore the homeostasis (Giorgi et al., 2015; Area-Gomez et al., 2019; Trebak and Kinet, 2019). However, the accumulation of Ca^2+^ in mitochondria favors oxidative damages, which can contribute to mitochondrial permeability and cell death (Tajeddine, 2016). In mitochondria, the oxidative stress is related to the partial reduction of molecular oxygen in the respiratory chain. Molecular oxygen is the final acceptor of electrons in the respiratory chain, and about 1–2% of molecular oxygen consumed in mitochondria is partially reduced to superoxide ion at the level of complexes I and III. Thus, the respiratory chain and other mitochondrial metabolic pathways produce superoxide ion that promotes oxidative stress after being converted into hydrogen peroxide, hydroxyl radical and peroxynitrite (Equations 1–3) (Murphy, 2009).

(1) $${\text{O}}^{-}-{\text{O}}^{\cdot }+{\text{O}}^{-}-{\text{O}}^{\cdot }\overset{\text{superoxide dismutase}}{\to }\text{HO}-\text{OH}+\text{O}=\text{O}$$

(2) $$\text{HO}-\text{OH}\overset{{\text{Fe}}^{\text{2+}}}{\to }{\text{HO}}^{-}+{\text{HO}}^{\cdot }$$

(3) $${\text{O}}^{-}-{\text{O}}^{\cdot }+{\text{N}}^{\cdot }=\text{O}\to \text{O}=\text{N}-\text{O}-\text{OH}$$

In addition to the radical species, the electronically excited species also contribute to mitochondrial oxidative damage. Oxidative processes promoted by the peroxidase activity of respiratory cytochromes on membrane lipids produce triplet carbonyls and singlet oxygen (Rodrigues et al., 2007). In mitochondria, cardiolipin plays a vital role in the proper functioning of the inner mitochondrial membrane proteins. Oxidative damages on mitochondrial lipids have been linked to a diversity of diseases such as muscular dystrophy, cancer, diabetes, premature aging, dementia, deafness, blindness, and male infertility (da Costa et al., 2016). The generation of pro-oxidant species in Ca^2+^- overloaded mitochondria are well-known inducers of MPTP opening. ANT, FOF1 ATPase, VDAC, and CypD are the mitochondrial proteins that are recognized as components and regulators of MPTP. There is a piece of evidence that pro-oxidant species can directly oxidize the MPTP components and regulators, but the role played by Ca^2+^ in this process is obscure. The protein CypD was postulated as a potential target for Ca^2+^ binding and regulation of MPTP opening. However, Nakagawa et al. (2005) and Baines et al. (2005) demonstrated that MPTP opening occurred in mitochondria from the heart, liver, and brain of mice lacking cyclophilin D (CypD^−/−^), although they required higher Ca^2+^ concentration than normal mitochondria. In a subsequent study, Giorgio et al. (2013) demonstrated the formation of Ca^2+^-sensitive MPTP by FOF1 ATPase incorporated in a lipid bilayer model. In a previous investigation, Grijalba et al. (1999) showed that Ca^2+^-CL complexation induces lipid packing changes and domains in submitochondrial particles (SMPs) and liposomes. The authors proposed that the Ca^2+^-induced in-plane rearrangements of lipids respond for the increased production of carbon-centered free radicals and membrane permeability in antimycin A-poisoned SMPs. Therefore, the studies above postulated that Ca^2+^-induced changes in lipid packing reverberate on the structure and aggregation of proteins involved in the membrane permeabilization. In the present study, it was demonstrated theoretically and experimentally that Ca^2+^ changes lipid packing and, even in the absence of proteins, the binding of Ca^2+^ favored lipid oxidation. Interestingly, oxidative effects of chemically generated free radicals and photochemically-generated singlet oxygen were similarly favored by Ca^2+^, at least concerning the formation of LOOH (lipid peroxide). The factors behind the different LOOH production kinetics determined in this paper are a challenging issue. Some aspects should be considered in trying to answer this question. For singlet oxygen generation, porphyrin TPPS4 was chosen precisely to prevent that the binding affinity of the photosensitizer to the membrane could interfer with oxidation kinetics. Therefore, as already shown in a previous study, with the use of TPPS4, most singlet oxygen is generated in bulk water, and membrane attack depends on the diffusion of the reactive species to the membrane. As Fe^2+^-citrate is also water-soluble under our experimental conditions, the oxidation by both singlet oxygen and Fe^2+^-citrate depend on the access of the reactive species to the lipid bilayer. Thus, the similar effects of Ca^2+^ on the CL oxidation kinetics by singlet oxygen and free radicals are consistent with changes in lipid packing (Chen et al., 2015). Cardiolipin oxidation by singlet oxygen in the presence of Ca^2+^ cannot be related to the arm-to-arm effect either (Yin and Zhu, 2012), as this mechanism is feasible to occur only in free radical-oxidized polyunsaturated fatty acids. Since the increase in the yield of cardiolipin peroxide in the presence of Ca^2+^ was similar for TOCL oxidation promoted by singlet oxygen and BBCL oxidation by free radicals, the significant favoring of the arm-to-arm mechanism by Ca^2+^ can be ruled out. The similarity of Ca^2+^effects observed for TOCL and BBCL also rules out the influence of the ion on the propagation of lipid peroxidation. Subsequent steps of lipid oxidation, including fragmentation, occur when the oxidizing agent is free radical acting on polyunsaturated fatty acids. Furthermore, lipid fragmentation products that are detected in biological membranes under oxidative stress were not observed in model lipid membranes under the action of singlet oxygen and free radicals (Kim et al., 2011). The significant effect of calcium on the kinetics of LOOH production in POPC membranes suggests that this ion also affects the packing of this phospholipid bilayers. However, the cause of the different kinetic profiles of LOOH production in POPC membranes to cardiolipin-containing membranes is a challenge that requires further studies. In the case of CL, both singlet oxygen and free radical oxidation depend on the access of reactive species to the interior of the bilayer. Therefore, the effect of calcium on the structure of cardiolipin predicted by theoretical studies is in line with the observed results. The pro-oxidant effect of Ca^2+^ that was non-specific for the oxidizing agent is consistent with changes in lipid packing that increase the membrane vulnerability to a diversity of oxidative attacks. Therefore, mitochondrial dysfunction associated with Ca^2+^ overload seems to be linked to distinct effects of Ca^2+^ on lipids and proteins. In the case of lipids, Ca^2+^ promotes changes in lipid packing that are enough to increase the susceptibility of membranes to the oxidation by free radicals and electronically excited species. Lipid oxidation favored by Ca^2+^ might have repercussions on protein structure and function and can occur associated with the effect of Ca^2+^ binding on proteins. Furthermore, in our DFT calculations, we observed that the effect of Ca^2+^ on CL is modulated by the interaction of the lipid head group with water.

## Materials and Methods

### Chemicals

The reagents 1-palmitoyl-2-oleoyl-sn-glycero-3-phosphocholine (POPC), tetraoleoyl cardiolipin (TOCL), and bovine brain cardiolipin (BBCL) were obtained from Avanti Polar Lipids, Inc. (Alabaster, AL, USA), TPPS4 (meso-tetrakis(4-sulfonatophenyl) porphyrin) was purchased from Sigma-Aldrich Corp. (St. Louis, MO, USA), Xylenol orange was purchased from Fluka Chemical Corp. (Ronkonkoma, NY, EUA). Glucose, sucrose, ammoniacal ferrous sulfate ((NH4)2Fe(SO4)2), chloroform, n-butanol, and ethanol were obtained from Synth (Labsynth Ltda., SP, Brazil), Deuterium oxide (D_2_O) was purchased from Cambridge Isotope Laboratories, Inc. (Andover, MA, EUA). The TPPS4 stock solution concentration was checked using the absorption coefficient ε at 412 nm = 5.30 × 10^5^ M^−1^ cm^−1^ (Kawai et al., 2014a).

### Giant Unilamellar Vesicles

Giant unilamellar vesicles of POPC/TOCL/TopFluorCL (90/9.5/0.5 mol%, respectively) were grown using the electroformation method (Riske et al., 2009; Haluska et al., 2012). The use of a lower cardiolipin percentage (10%) in GUVs than in LUVs (20%) is necessary for the better stability of the vesicles. Briefly, 40 μL of a mixture of lipids in the concentration of 2.5 mM dissolved in chloroform solution was spread on two conductive glass plates (coated with indium tin oxide). The apparatus was positioned with the conductive sides facing each other and separated by a 2-mm-thick Teflon frame. In the following, the electro swelling chamber was filled with a 0.2 M sucrose solution. The apparatus was connected to an alternating power generator at 1 V with a 10 Hz frequency for 2 h at 25°C. The vesicle suspension was removed from the chamber and diluted ~6 times into 0.2 M glucose solution. The optical sugar asymmetry between the interior and exterior of the giant vesicles created differences in the density and refractive index between the sucrose and glucose solutions; the vesicles were, therefore, stabilized at the bottom of the chamber by the action of gravity, and had the best contrast when observed under phase-contrast microscopy. Observation of the giant vesicles was performed under an inverted microscope, the Zeiss Observer A1 (Carl Zeiss, Jena, Germany), equipped with a 40X objective. Images were obtained using an AxioCam R3 digital camera (Carl Zeiss, Jena, Germany). The vesicle suspension was placed in a special chamber, which consists of an 8-mm-thick Teflon frame between two glass plates, through which observation was possible.

### Large Unilamellar Vesicles (LUVs)

Lipids, POPC/CL (80/20 mol%), POPC (100%), and CL (100%) were initially dissolved in chloroform and the solvent evaporated under a flow of N_2_. The lipid film was kept under reduced pressure for at least 2 h, after which it was hydrated by adding a 0.2 M glucose, pH 6.5, at room temperature. Unilamellar liposomes were obtained by extrusion of hydrated lipid dispersions in an Avanti Mini-Extruder, acquired from Avanti Polar Lipids, Inc. (Alabaster, AL, USA). Multilamellar liposomes were subjected to 11 passes through two polycarbonate filters (100 nm pore size, Nucleopore, Pleasanton, CA, USA) installed in tandem. Liposome solutions were diluted with the buffer to the final lipid concentration of 250 μM.

### Generation of Singlet Oxygen and Free Radicals

Where indicated, porphyrins were excited by irradiation with a white LED array. The LED array emits 200 μW/cm2 in 532 nm measured by FieldMate PowerMeter (Coherent Inc.). Lipid photo-oxidation was promoted by 10 μM of TPPS4. For the oxidation promoted by free radical generated by Fe^2+^/citrate polyunsaturated BBCL was used and incubated in the presence of 50 μM Fe^2+^/citrate (Pessoto et al., 2009). Data are presented as the mean ± s.e.m. of three experiments with different liposome preparations. Where indicated, lipid oxidation of lipids in LUVs was carried out using POPC/TOCL (80/20 mol%, respectively), POPC 100%, TOCL 100%, and BBCL 100% in a solution of 0.2 M of glucose, pH 6.5. The presence of glucose in the medium has the objective to reproduce the same conditions of the experiments with GUVs.

### Lipid Oxidation Assays

Lipid oxidation was evaluated with lipid peroxide (LOOH) production. Lipid hydroperoxide concentration was determined by the oxidation of Fe(II) in the presence of xylenol orange: aliquots of the sample (10 μL) were extracted from incubation at room temperature and mixed with 890 μL of Milli-Q^®^ water and 100 μL of hydroperoxide reagent that contained 2.5 mM xylenol orange, 2.5 mM (NH_4_)_2_Fe(SO_4_)_2_, and 1.15 M HClO_4_ in Milli-Q^®^ water. The sample was rested for 30 min at room temperature. The oxidation of Fe(II) by LOOH generates Fe(III), which reacts with xylenol orange and is converted to the colored product that absorbs at 560 nm. LOOH concentration was calculated by using ε = 560 nm = 6.45 × 10^4^ M^−1^cm^−1^. TBARS was determined at 535 nm after reaction with thiobarbituric acid (TBA), using the molar extinction coefficient 1.56 × 10^5^ M^−1^cm^−1^.

### Electronic Absorption Spectra Measurements

The electronic absorption spectra of porphyrins were measured by using a Varian Cary 50, Varian Inc. (CA, United States) spectrometer. The spectral resolution for wavelength scan was 0.5 nm. The optical path length was 1 cm for the LOOH dosages, and 0.1 cm for the porphyrin spectra measurements and quantification.

### FTIR Spectroscopy

An *in situ* attenuated total reflectance-Fourier transform infrared (ATR-FTIR) technique (Mudunkotuwa et al., 2014) has been used to study the protonation of 10 mM cardiolipin vesicles at pH 2 and 6 (Hielscher et al., 2009). Infrared spectra were recorded using a Varian 640-IR Spectrometer fitted with a DLaTGS detector and a PIKE MIRacle™ single reflection horizontal ATR accessory with around 1.8 mm of sampling área diamond/ZnSe crystal and 45 degrees angle of incidence. A series of spectra were measured corresponding to each sample conditions after 15 μL of the sample had been dropped in contact with the crystal and let drying at room temperature (20°C ± 1) for 30–40 min. These thin films as shaped have enough optically sampled surface area. The surface area allows for good quality infrared spectra to be obtained from a single internal reflection. The reference spectrum was the crystal. All spectra were calculated from 64 scans at 4 cm^−1^ resolution and were not corrected for the wavelength dependence of absorbances.

### Theoretical Calculations

The *ab initio* DFT calculations were performed with the code Car-Parrinello Molecular Dynamic (CPMD), using Troullier-Martins pseudopotentials with the inclusion of London dispersion forces (DCACPs) (Kohn and Sham, 1965; Troullier and Martins, 1991; Von Lilienfeld et al., 2005)^1^. All molecular structures were fully relaxed until the forces on which atom was <0.02 eV/Å. The Kohn-Sham orbitals were expanded in a set of plane waves with the cutoff energy of 110 Ry (Furmanchuk et al., 2010; Rössle et al., 2010; Bernardi and Pascutti, 2012; Payal et al., 2012; Dreyer et al., 2013). In all phospholipids, the interaction parameter in the z-direction (CL frontal structure) was set to 5.6 Å. This value was previously minimized for a range of 4–6.4 Å (z-axis).

## Data Availability Statement

The raw data supporting the conclusions of this manuscript will be made available by the authors, without undue reservation, to any qualified researcher.

## Author Contributions

IN-C conceived experiments. ÉM and JA-C conducted the experiments and analyzed part of the results. ID and JA performed theoretical calculations. IN-C and JA analyzed the results and wrote the manuscript. CK performed GUV production and images. AB performed FTIR experiments. All authors reviewed the manuscript.

### Conflict of Interest

The authors declare that the research was conducted in the absence of any commercial or financial relationships that could be construed as a potential conflict of interest.

## Footnotes

^1^CPMD, Copyright IBM Corp 1990–2015, Copyright MPI für Festkörperforschung Stuttgart 1997–2001. Available online at: https://www.cpmd.org/wordpress/index.php/the-code/ (accessed January 9, 2019).
